# The resource availability hypothesis (RAH) and cross-cultural patterns: which one explains West African *Cochlospermum* species’ uses in Benin?

**DOI:** 10.1186/s13002-022-00555-3

**Published:** 2022-08-23

**Authors:** Gnimansou Abraham Favi, Gbèwonmèdéa Hospice Dassou, Donald Djidohokpin, Jéronime Marie-Ange Sènamie Ouachinou, Chabi Ghyslain Kpétikou, Eutiche Gbedolo, Alain Anagonou, Noelia Hidalgo-Triana, Aristide Cossi Adomou

**Affiliations:** 1 Laboratory of Botany and Plant Ecology, Faculty of Sciences and Techniques, University of Abomey-Calavi, 01 BP 4521 Cotonou, Republic of Benin; 2 Departamento de Biología Vegetal (Botánica), Facultad de Ciencias, Universidad de Málaga, 29010 Malaga, Spain

**Keywords:** *Cochlospermum* spp., Wild edible plants, Sustainability, Availability hypothesis, Cultural importance

## Abstract

**Background:**

*Cochlospermum tinctorium* and *C. planchonii* are two wide edible plants of sub-Saharan countries, e.g., Benin, widely used as food, medicine, dye, handicraft, etc. Unfortunately, the uncontrolled harvest of their rootstocks exposes them to local extension risk. To enhance knowledge on the determinants of their uses in Benin, this study aimed to (i) assess the use forms, use values, abundance and perceived spatiotemporal dynamics, (ii) determine how does local abundance or cultural patterns affect the use of *Cochlospermum* species, and (iii) assess local harvesting modes and conservation management practices.

**Methods:**

In total, 756 Dialog Partners through 27 ethnic groups were questioned countrywide using semi-structured interviews. Questions refer to local taxonomy, specific uses, organs sought, harvesting modes and local conservation strategies; afterward, local abundance of each species was assessed. Ethnobotanical indicators were analyzed through citation frequencies to obtain quantitative data. Comparison tests and statistical analyses were performed using R program.

**Results:**

*C. tinctorium* and *C. planchonii* are locally well known and involved into 83 specific uses, grouped into ten categories of which medicinal use was the main. The use values of *C. planchonii* (0.10 ± 0.19) and *C. tinctorium* (0.23 ± 0.20) varied significantly between the ethnic groups, and only *C. tinctorium* showed index of commercially value above 75% for some ethnic groups. The current abundance, about 84 and 97 tufts.ha^−1^, respectively, for *C. planchonii* and *C. tinctorium* was perceived with a decline of 81.09% (*C. tinctorium*) and 73.7% (*C. planchonii*) of informants. Moreover, the Spearman’s correlation and Kruskal–Wallis tests performed revealed that the use values of *C. tinctorium* and *C. planchonii* were significantly correlated on the one hand with their local abundance and on the other hand with the investigated ethnic groups. About 42.3% of women produced the powder as principal activity, while more than 57% produced it mainly at the end of farm work. However, the conservation management was practiced by very few informants and consisted of partial harvesting of rootstocks (41.8%, only in southern Benin), and fallowing of harvesting areas (3.97%, only in northern Benin).

**Conclusion:**

Facing the declining abundance and increasing market demand for rootstock powder of *Cochlospermum* species, existing local conservation strategies should be promoted and the domestication process should be initiated for sustainable management of these important wild edible plants before these important resources disappear completely in the wild.

## Background

Worldwide, rural communities mainly rely on wild edible plants (WEPs) to manage their daily subsistence (food, physical and health needs), but also their cultural and economic needs [1]. In recent decades, ethnobotanical studies have analyzed the dynamics of people–plant relationships under different perspectives [2]. Understanding the dynamics of traditional knowledge of people could provide significant implications, notably the checklist of plants used by people, identification of their phytochemical and biological properties, optimization of their uses, and exploration of new insights into the impacts of usages on their conservation status [3]. On the other hand, understanding how and why people select plants for a wide range of uses has been in response to a repeated call for theory-inspired and hypothesis-driven research to improve the rigor of ethnobotany as a discipline [4]. Thus, among the major hypotheses, the resource availability hypothesis (RAH) has been highlighted, which states that a given plant is used because of its accessibility or local abundance [4–6]. This hypothesis attempts to elucidate why people use a given species more than others and especially whether spatial distribution patterns of species relate to patterns of use [7].

RAH was adapted from the ecological apparency hypothesis initially proposed by Feeny [8] and Rhoades and Cates [9] and first implemented in herbivore studies. In this way, Phillips and Gentry [10, 11] have developed a quantitative measure, the use value (VU), to measure the relative importance of given plant species to a community on the basis of its use reports. RAH states that the most available species tend to have the highest use value, making this relationship directly proportional [12]. It has been tested in several works by correlating the local abundance or dominance of plant species with their use values (UV) in many distinct phytogeographical regions [4, 12–18]. For instance, consistent supports were found for the availability hypothesis according to Hart et al. [19] for Ecuadorian pharmacopeia, and [20] in Semiarid Region of Brazil. By this, the authors were looking for not only to understand this relation, but also to set standards for the demand of certain groups of species through the test of the ecological apparency hypothesis [20]. In Benin, research studies have provided an overview of the threats on some plant species due to their use values across various ethnic groups in rural communities as the case of *Borassus aethiopum* Mart. [21], *Afzelia africana* Smith ex Pers*.* and *Khaya senegalensis* (Desv.) A. Juss. [22].

In this study, we used the case of *Cochlospermum* species (*Cochlospermum planchonii* Hook.f. ex Planch. and *Cochlospermum tinctorium* Perrier ex A. Rich.) to test the resources availability and cross-cultural patterns hypotheses. Indeed, *Cochlospermum* species (Bixaceae, previously Cochlospermaceae) are important wild edible and multipurpose plants, used in West Africa for a wide range of purposes such as food, folk medicine, dye and in handicraft [23, 24]. The genus includes *C. tinctorium* and *C. planchonii*, with a possible occurrence of a third possible species, namely *C. intermedium*, which has not yet been formally identified [25]. *C. intermedium* generally spreads where the occurrences of *C. tinctorium* and *C. planchonii* overlap [26, 27], as in the Sudanian and Guineo-Sudanian zones of Benin. In this study, *C. tinctorium* and *C. planchonii* will be focused since they are commonly known and mentioned in the literature [23, 28]. In Benin, with regards to their roles as sources of food and income generation notably during periods of food shortage and in health care, *C. tinctorium* and *C. planchonii* have been reported among the 140 WEPs identified [23, 28, 29].

Unfortunately, they have still not been investigated and little attention has been given to them notably concerning their conservation status [30]. Also, there is a need for further documentation of the complexity of the plants use value, distribution, anthropogenic factors threatening the sustainability and conservation status in different ecological zones [30, 31]. Based on the information above mentioned, to enhance the current knowledge on the *Cochlospermum* species’ uses and ensure their sustainable management, it appeared important to give insight on how their use values are affected by the local abundance and how they vary within the ethnic groups of Benin. Therefore, the following three specific objectives were addressed: (i) to assess the use forms, use values, local abundance and perceived spatiotemporal dynamics across the ethnic groups and phytogeographical zones, (ii) to determine how local abundance (availability) or cross-cultural patterns affects the use of *Cochlospermum* species, and (iii) to assess local harvesting modes and conservation management practices.

## Methods

### Research area

**Fig. 1:**
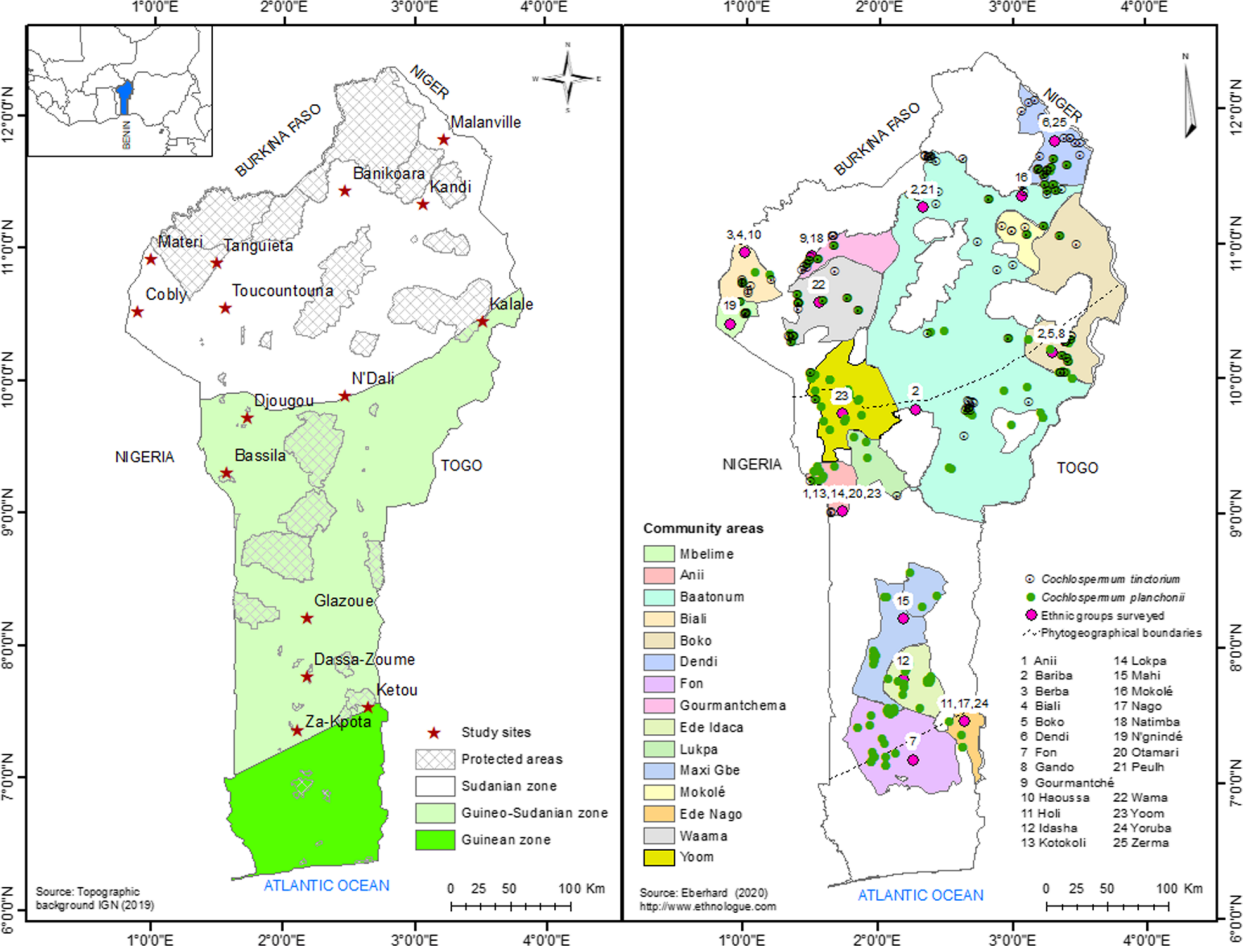
Study area showing surveyed municipalities (at left) chosen by overlaying ethnic groups and species geographical distribution (at right). Species names (blue), plant parts used (green), use categories (orange), and specific uses (gray)

This study was conducted in the Republic of Benin, located in West Africa between latitudes 6°10′N-12°25′N and longitudes 0°40′E-3°45′E (Fig. 1). It covers an area of 114,763 Km^2^, with resident population estimated at 11,496,140 inhabitants in 2018. Nine major ethnic groups and allies are found, including Fon and allies (38%), Adja and allies (15%), Yoruba and allies (12%), Bariba and allies (10%), Peulh (9%), Gua or Otamari and allies (6%), Yoa and Lokpa and allies (4%), Dendi and allies (3%) and others (3%).

Three phytogeographical zones are distinguished, namely the Sudanian zone (SZ) in north, the Sudano-Guinean zone (SG) in the center and the Guineo-Congolian zone (GC) in the south [25]. The vegetation, hosting a rich biological diversity including *Cochlospermum* species, is essentially made of fallow and small dense forest area in the Guinean-Congolian zone; the transition zone, namely Sudano-Guinean zone is well represented by mosaics of savannahs, dry forests and riparian forests, while the north is dominated by a patchwork of woodlands and savannahs with belts of riparian forests along rivers [32]. Plant and animal resources are conserved through 49 protected areas across the country (Fig. 1). The agriculture remains the main livelihood activity carried out by the people [33]. The local communities are well known for their traditional beliefs and use of plants for subsistence, worsening the main threats for plant species [34].

### Studied species

*C. tinctorium* and *C. planchonii* are two wild multipurpose plant species that spread across the dry regions of West Africa [26, 35]. As previously described in [24, 36], they are both xeromorphic and subshrubs, commonly used by rural communities for various purposes such as food, medicine for humans and animals, dye and handicraft [24, 35]. Their woody subterranean rootstocks [37, 38] are the main parts of these species used. *C. planchonii* occurs from Senegal eastwards to Chad, in savannah and forest savannah mosaic and fallows [35], while *C. tinctorium* extends from eastern to southwestern Sudan and northwestern Uganda in dry savannah and prefers devastated, rocky and annually burnt areas [39, 40]. In Benin, *C. tinctorium* spreads across the Sudanian region and scarcely in the Sudano-Guinean region in the savannah woodlands and grasslands [25]. However, *C. planchonii* extends countrywide, mainly in the Sudanian and Sudano-Guinean regions and scarcely in the Guineo-Congolian region. Both species are more detailed in [24].

*C. tinctorium* produce annual leafy shoots in the rainy season, reaching 80 cm tall [39] and 1.5–2.5 m tall for *C. planchonii* [25]. *C. planchonii* flowers appear toward the end of the rainy seasons and fruits appear 1–2 months after, while *C. tinctorium* produce flowers in the dry season after the bushfires, and the fruits are ripe about 1 month after flowering [25, 39]. *C. planchonii* and *C. tinctorium* as well as all other *Cochlospermum* species grow as common weeds of cultivation, reproducing naturally from seeds [41].

### Ethics statement

The research protocol adopted in this study followed the components of the Ethical Code of the International Society of Ethnobiology [42] and was approved by the Scientific Ethic Committee of the Graduate School of Life and Earth Sciences (EDSVT) of the University of Abomey-Calavi (UAC) under the referral code: N° 117-15/EDSVT/FAST/UAC. The aims of the study, after having been carefully explained to the local leaders and informants, received oral consent, and interviews were conducted in anonymity and fully mindful and respectful manner [42]. Oral consent was preferred to written consent because of the largely illiterate populations we worked with.

### Sampling design and ethnobotanical surveys

#### Sites and partners of study

The sites of study were delimited by overlaying the map of distribution of the species, across the three bioclimatic zones [36], with the map showing the repartition of different ethnic groups in the country sourced from the “Ethnologue: Languages of the World” dataset [43]. The generated map shows 15 polygons which represent the areas of overlap between the species distribution and ethnic areas (Fig. 1). From all the communities covered by the areas of overlap in each bioclimatic zone, while several ethnic groups are found in an area, we selected municipalities based on the dominant ethnic groups.

Overall, 756 DPs were interviewed in the study, of which 359 men and 397 women, distributed through 27 ethnic groups, and 15 municipalities. They were between 17 and 92 years old with an average of 43 years old, and the majority (47.35%) were 36–50 years old. The PDs comprised 34% of farmers, 29% of traditional healers, 25% of roots powder sellers, elder people and local leaders (8 and 4%, respectively). They are over the three bioclimatic zones as follows: 401 DPs (53.04%) in the Sudanian zone, 299 DPs (39.55%) in Sudano-Guinean zone and 56 DPs (7.41%) in Guineo-Congolian zone (Table 1).

**Table 1:** Ethnobotanical indicators analyzed through the dataset

Questions	Item’s groups	Indexes	Formulas
Use categories	Medicinal, food, handicraft, construction, fodder, dye, magic, fuel, veterinary, cosmetic	Use value	$$UVs = \sum {UV_{{is}} } {\raise0.7ex\hbox{${{\text{x}}^{1} }$} \!\mathord{\left/ {\vphantom {{{\text{x}}^{1} } {N_{s} }}}\right.\kern-\nulldelimiterspace} \!\lower0.7ex\hbox{${N_{s} }$}}$$ *UVis*: the use value of the species *s* for an informant *i* and *Ns* the total number of persons interviewed for the species *s* $$UV_{{is}} = \sum {U_{{is}} } {\raise0.7ex\hbox{${{\text{x}}^{1} }$} \!\mathord{\left/ {\vphantom {{{\text{x}}^{1} } {N_{s} }}}\right.\kern-\nulldelimiterspace} \!\lower0.7ex\hbox{${N_{s} }$}}$$ *Ui*: the number of uses mentioned by each informant for species, n: the total number of informants. [44]
Organs exploited	Rootstock, root bark, leaf, stem, stem's bark, fruit, fibber, flower, seed	Index value of organ (IVO)	$$IVO = \sum {N_{{ui}} } {\raise0.7ex\hbox{${{\text{x}}^{1} }$} \!\mathord{\left/ {\vphantom {{{\text{x}}^{1} } {N_{{tu}} }}}\right.\kern-\nulldelimiterspace} \!\lower0.7ex\hbox{${N_{{tu}} }$}}$$ *Nui*: the number of use patterns of each organ quoted by informants *i* and *Ntu* the total number of uses of all organs quoted by the informants *N*. [45]
Commercial value	Yes/no	Index of commercial value (ICV)	$ICV = I_{p} /I \times 100\%$ *Ip*: the number of informants answering positively to sell the species plant parts and *I*: the total number of informants questioned (adapted from Lozano et al. [13])
Species abundance		Density (*N*_*i*_)	$Ni = n_{i} /S$ *Ni*, the abundance in the plot *i*; *ni*, the number of individuals in the plot *i,*and *S* the area (1 ha)
Species dynamic	Increase, decrease, stable	Citation frequency (FC)	$FC = N_{p} /N_{t} x100\%$ *Np*: the number of times a particular response was mentioned and *N*_*t*_: the total number of informants questioned (adapted from Faruque et al. [46])
Harvesting modes	Axe, hoe, machete		
Conservation management practices	Seeding, assisted natural regeneration, partial harvest, fallowing areas, weeding protection		

#### Interview process

The data related to the local knowledge and uses on *Cochlospermum* species were collected through an ethnobotanical survey. The field survey was carried out from October 2018 to April 2019, and interviews were conducted in the local language of the PDs [47]. For this, we used the semi-structured questionnaire, dried specimens (herbarium), and photographs as materials for recognition and distinction of *Cochlospermum* species by PDs [48].

On the one hand, the folk name(s), their meaning and local criteria based on the morphological traits used to distinguish both species were recorded. On the other hand, PDs listed the specific uses of each species, the purposes of use, and the plant parts sought. The local dynamic perceived, the threats and the causes were also noted. Informants are also asked if they use the root powder for commercial purposes. To minimize as far as possible interference of other people during the interviews, individual meetings were held according to a specific schedule [10]. Furthermore, informants were asked to identify in the wild the species occurring in their area where they harvest the organs [13].

#### Vegetation sampling

To test the availability hypothesis, the density of each species was assessed on-site where species were collected (forests, savannahs and fallows). In the surroundings of each village where ethnobotanical survey has been done, major vegetation was identified with the help of the Dialog Partners. These areas constituted the site where local communities harvest *Cochlospermum* species parts and were chosen based on the majority agreement of informants. The abundance data were collected within 100 plots of 100 × 100 m each. Countrywide, 27 areas were selected and sampled during the field works. For each *Cochlospermum* species and by plot, the number of tufts, considered as individuals, was counted. According to the geographical distribution of each *Cochlospermum* species in the study area, *C. tinctorium* was sampled in the Sudanian and Sudano-Guinean zones, while *C. planchonii* was sampled countrywide (Guineo-Congolian, Sudano-Guinean and Sudanian zones).

### Data analysis

#### Local knowledge

Dialog Partners’ responses were prior grouped into answers’ groups according to their similarities. The ethnobotanical indicators analyzed through the dataset were the citation frequencies, the species use values, species density, index of commercial value and the index value of organs (Table 1). For each question section, citation frequency was calculated for each response level for a given species, by converting the presence–absence matrix into quantitative data. It is defined as the proportion between the number of positive answers (number of citations) for each questionnaire item and the total number of informants [46]. To determine the species’ uses, the recorded usages were arranged by use category and the frequency of each use category was computed. Ten categories were retained, based on specialized literature [10, 14, 24]. In addition, specific affections treated by each species were ranged into ten categories, corresponding to various body systems [46, 49], and ailments non-specific to a given system were considered as general health care.

The usefulness of each *Cochlospermum* species using the use values (UVs) was assessed [44] and compared between ethnic groups through the paired t test to determine for which purposes ethnic groups used mostly a given species. The index values of organs (IVO) and the index of commercial value (ICV) were expressed, respectively, according to Balima et al. [45] and Lozano et al. [13] (Table 2). A given species was considered as commercially important when sellers were more than 75% of whole informants. The Spearman’s correlation analysis was performed to assess the correlation between the commercial values of each *Cochlospermum* species in an ethnic group [50].

**Table 2:** Folk taxonomy of *C. tinctorium* and *C. planchonii*

Ethnic groups	*C. tinctorium*	*C. planchonii*
Anii	–	*Abuburoumey*^1^
Bariba	*Kpadou, Kpararou*	*Tòòri, Tòòra*^2^
Berba	*Tchotcho'ndaha*	*Tchotcho'nihou*^1^
Biali	*Tchotchon'da*	*Tchotcho'nihou*
Boko	*Kpaà*	*Kounwó, kòli, koó*
Dendi	*Kpata*	*Kpata*
Fon	–	*Avokanfoun tchéké, Alovi aton*
Gando	*Djaloudji*	*Djaloudji*
Gourmantché	*Lissaya'djaga,*	*Lissaya'nigou*^3^*, Tissa'ndi*
Haoussa	*Kouata*	*Balidjè*
Holi	–	*Gbétoun*
Idaasha	–	*Tchôôri*
Kotokoli		*Kouloumbokou*
Lokpa	*Djèhindjé*	*Djèhindjé*
Mahi	–	*Kpôdouyin*
Mokolé	*Kpata*	*Kitigbo*
Nagot	–	*Gbètou*^4^
Natimba	*Souinhinri*	*Souinhinri*^1^
N'gnindé	*Dissondé*	*Dissondé*
Peulh	*Djaloudji*	*Djaloudji*
Wama	*Boussorobu’dafa*	*Boussorobu’nibou*^5^
Yoom	*Toutouworkô*	*Toutouworkô*
Zerma	*Kpata*	*Kpata*
Otamari	–	*Dissondi*^6^
Yoruba	–	*Gbèhoutou*^*4*^*, Fèroun*

The exponents of folk names indicate the meaning of the corresponding names (1: plant which cures; 2: plant used in child baptism (root powder is used in sauce preparation during this ceremony); 3: plant that alleviates diseases; 4: plant with therapeutic substance; 5: dog meat flavor (to destroy ailments in dog meat); 6: wild tomatoes)

The harvesting modes and conservation management practices developed by informants were assessed using citation frequency. The conservation practices were compared between the phytogeographical zones. The matrix of use value of all categories and the ethnic groups was submitted to principal component analysis (PCA) to determine the links between both the factors. All these statistical analyses were performed using R software 1.4.1103 [51].

#### Species abundance

In each plot, *Cochlospermum* species density was computed using the density equation (Ni) (Table 2).

#### Relation between use value, local abundance and ethnic groups

To test the resource availability hypothesis (RAH), the use value (UV) of each species in given ethnic group was combined with the density (abundance) of this species recorded in the corresponding area [15, 52]. This analysis was performed using the Spearman’s correlation coefficient [50]. Then, the variation of the UV of each *Cochlospermum* species through the ethnic groups was analyzed using the Kruskal—Wallis test, considered as statistically significant for *p* values < 0.05.

## Results

### Folk taxonomy

*Cochlospermum* species were identified through different names according to the ethnic groups (Table 3). Overall, thirty-six vernacular names were recorded across the ethnic groups, with six names common to both *Cochlospermum* species, fourteen names specific to *C. tinctorium* and twenty-eight specifics to *C. planchonii*. 56% and 67% of names were, respectively, reported for *C. tinctorium* (e.g., *Tchotcho'ndaha* and *Boussorobu’dafa*) and *C. planchonii* (e.g., *Avokanfoun tchéké*, *Lissaya'nigou, Tissa'ndi*); these were composed of two words, while 44% and 33% of them were, respectively, made of single word. Also, homonymy case in vernacular name was noted in Dendi and Zerma ethnic groups, naming both species *Kpata*. Moreover, six out of the thirty-six vernacular names recorded referred essentially to food and medicinal uses criteria. Empty boxes mean the absence of the species in the corresponding locality.

**Table 3:** Sociodemographic parameters of informants

Bioclimatic zones (DPs)	Ethnic communities	Study municipalities	Main ethnic groups	Sample size	Men	Women	Ages		
							17–35	36–50	> 50
Sudanian 401 DPs (53.04%)	Bariba	Banikoara	BaribaB, Peulh	59	31	28	26	29	4
	Mbelime	Cobly	N'gnindé	29	16	13	9	14	6
	Mokolé	Kandi	Mokolé	30	12	18	13	10	7
	Dendi	Malanville	Dendi, Zerma	72	24	48	29	34	9
	Biali	Matéri	Berba, Biali, Haoussa	89	40	49	30	49	10
	Gourmantchema	Tanguiéta	Gourmantché, Natimba	59	28	31	22	29	8
	Waama	Toucountouna	Wama	63	27	36	25	27	11
Sudano-Guinean 299 DPs (39.55%)	Anii	Bassila	Anii, Kotokoli, Otamari	79	38	41	20	40	19
	Yom	Djougou	Yoom	37	14	19	15	18	7
	Lokpa	Djougou	Lokpa	23	12	15	11	8	1
	Ede Idaca	Dassa-Zoumè	Idasha	16	11	5	4	9	3
	Maxi Gbe	Glazoué	Mahi	19	12	7	5	9	5
	Boko	Kalalé	Bariba, Boko, Gando	76	28	48	33	28	15
	Ede Nago	Kétou	Nago	21	14	7	5	11	5
	Bariba	N'Dali	Bariba	28	15	13	10	16	2
Guineo-Congolian 56 DPs (7%)	Ede Nago	Kétou	Holi, Yoruba	60	40	20	15	30	15
	Fon	Zakpota	Fon	17	11	6	4	8	5
Total		-	-	756	359	397	271	358	127

Bariba ethnic group surveyed in Banikoara, Kalalé and N’Dali municipalities are, respectively, named BaribaB, BaribaK and BaribaN; Wama ethnic group surveyed in Tchakalakou and Moussitingou villages are, respectively, named WamaT and WamaM

### Traditional uses and species local abundance

#### Specific uses and plant parts exploited

**Fig. 2:**
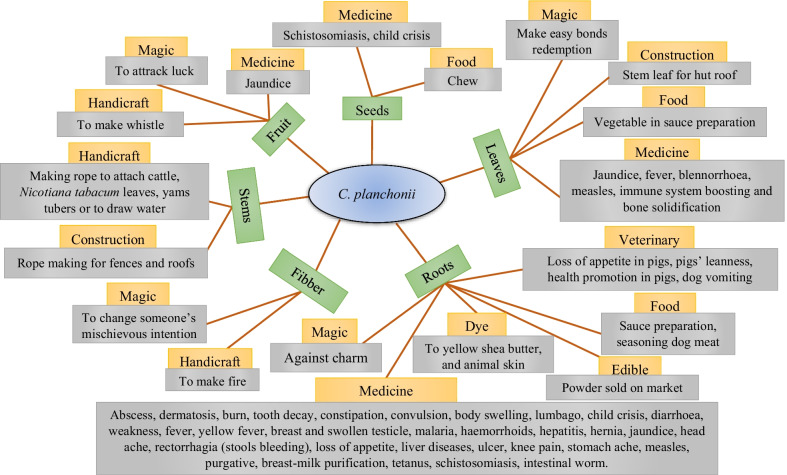
Traditional use forms and plant parts of *C. planchonii*. Species names (blue), plant parts used (green), use categories (orange) and specific uses (gray)

**Fig. 3:**
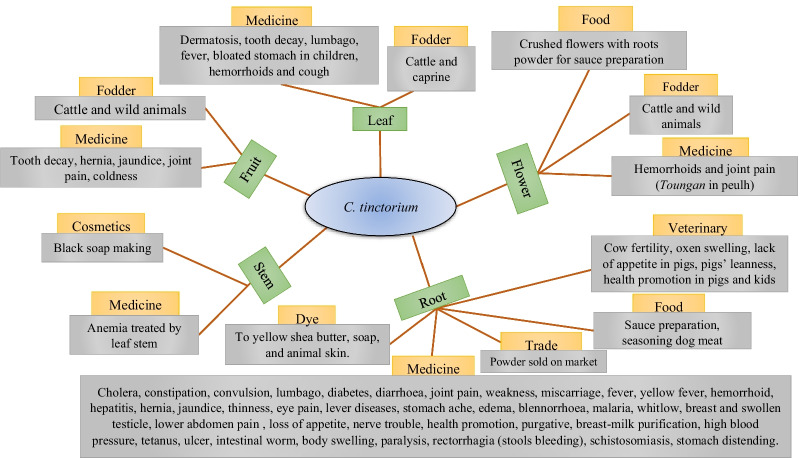
Traditional use forms and plant parts of *C. tinctorium*

*Cochlospermum* species were reported in 83 specific uses, of which 57 were specific to *C. planchonii*, 61 specific to *C. tinctorium*, and 35 common to both species. These uses were grouped into ten (use categories for *C. tinctorium* and seven for *C. planchonii*. All the use reports were summarized for *C. planchonii* (Fig. 2) and *C. tinctorium* (Fig. 3). Broadly, the use values (UV) expressed through the means and standard deviation varied according to the species, with UV = 0.10 ± 0.19 for *C. planchonii* (Table 4) and 0.23 ± 0.20 for *C. tinctorium* (Table 5). Considering the phytogeographical zones, Sudanian zone (0.12 ± 1.10) exhibited the highest use value, followed by the Sudano-Guinean (0.09 ± 0.97) and Guineo-Congolian (0.06 ± 1.00) zones for *C. planchonii*, while the use value for *C. tinctorium* in the Sudanian zone was 0.24 ± 1.27, followed by Sudano-Guinean zone (0.20 ± 1.27).

**Table 4:** Use values, density and commercial value of *C. planchonii*

Ethnic groups	UV ± sd	Ni	UVc ± sd									ICV
			Medicinal	Food	Handicraft	Construction	Fodder	Dye	Magic	Fuel	Veterinary	
Anii	0.10 ± 1.05	71	19 ± 0.65^a^	9 ± 0.50^c^	6.5 ± 0.48^b^	2.5 ± 0^c^						6.45
BaribaB	0.10 ± 1.14	79	13.5 ± 0.70^b^	6.5 ± 0.27^c^	8 ± 0.49^b^	4.5 ± 0.38^b^	3 ± 0^b^			3 ± 0^a^		48.72
BaribaK	0.09 ± 0.93	89	10 ± 0.78^c^	7 ± 0.28^c^	6 ± 0.42^b^	3 ± 0.38^c^	3 ± 0.38^b^	3 ± 0^b^		2 ± 0^b^		53.33
BaribaN	0.11 ± 0.98	79	16.5 ± 0.78^a^	8 ± 0.36^c^	8.5 ± 0.25^b^	5 ± 0^b^		3 ± 0^b^				28.57
Berba	0.09 ± 1.06	101	14.5 ± 0.69^b^	10 ± 0.47^b^	9 ± 0.43^a^							51.61
Biali	0.18 ± 1.26	79	26.5 ± 0.92^a^	17 ± 0.47^a^	8.5 ± 0.48^b^	4 ± 0.35^b^	5 ± 0^a^	3.5 ± 0.35^b^				48.28
Boko	0.09 ± 1.21	97	9.5 ± 0.99^c^	6.5 ± 0^c^	7 ± 0.28^b^	4.5 ± 0.40^b^	3 ± 0.46^b^	4.5 ± 0.32^b^				44.83
Dendi	0.08 ± 0.76	100	10 ± 0.48^c^	5.5 ± 0^c^	9 ± 0.65^a^	7 ± 0^a^						41.94
Fon	0.06 ± 1.07	41	7.5 ± 0.53^c^		8 ± 0.44^b^				5.5 ± 0.67^a^			
Gando	0.07 ± 0.89	89	10.5 ± 0.48^c^	4.5 ± 0.32^c^	2.5 ± 0^c^	2.5 ± 0^c^	2 ± 0^b^	1.5 ± 0^c^			5 ± 0.53^b^	52.94
Gourmantché	0.12 ± 1.00	91	9.5 ± 0.69^c^	2.5 ± 0.52^c^	10 ± 0.53^a^	6.5 ± 0.41^b^	2.5 ± 0^b^	4 ± 0^b^	5.5 ± 0.67^a^		2.5 ± 0^c^	40.63
Haoussa	0.12 ± 0.93	81	12.5 ± 0.84^b^	13 ± 0.39^b^	8.5 ± 0.64^b^	8 ± 0.30^a^						37.93
Holi	0.08 ± 1.08	71	13 ± 0.71^b^	4 ± 0^c^	6 ± 0^b^				6.5 ± 0.67^a^			4
Idasha	0.04 ± 0.89	48	10.5 ± 0.77^c^		5.5 ± 0^b^							
Kotokoli	0.13 ± 1.19	91	22 ± 0.75^a^	12.5 ± 0.44^b^	8.5 ± 0.58^b^	4 ± 0^b^			4.5 ± 0.75^a^			41.67
Lokpa	0.10 ± 0.83	99	10.5 ± 0.64^b^	11 ± 0.37^b^	8.5 ± 0.44^b^	6 ± 0.30^b^						
Mahi	0.06 ± 0.85	64	14.5 ± 0.80^b^		6.5 ± 0^b^							
Mokolé	0.17 ± 1.34	97	17.5 ± 1.10^a^	22.5 ± 0.67^a^	9 ± 0.67^a^	4 ± 0^b^	2 ± 0.45^b^	3.5 ± 0^b^	2.5 ± 0.75^b^	3 ± 0^a^		66.67
Nago	0.07 ± 1.08	43	16.5 ± 0.81^a^		6 ± 0^b^				4.5 ± 0^a^			
Natimba	0.14 ± 1.41	93	13 ± 0.77^b^	11 ± 0.85^b^	11 ± 0.69^a^	8 ± 0.55^a^	3.5 ± 0^b^	3.5 ± 0^b^	2.5 ± 0^b^			40.74
N'gnindé	0.17 ± 1.35	101	14 ± 1.14^b^	13 ± 0.68^b^	13 ± 0.60^a^	11 ± 0.51^a^		6.5 ± 0^a^	6.5 ± 0.40^a^			41.38
Otamari	0.08 ± 0.92	96	12 ± 0.45^b^	8.5 ± 0.48^c^	5.5 ± 0^b^				2 ± 0^b^			25.00
Peulh	0.08 ± 0.83	91	8.5 ± 0.48^c^	4 ± 0^c^	3.5 ± 0^c^	2 ± 0^c^	3 ± 0^b^				7.5 ± 0.50^a^	3
WamaM	0.11 ± 1.16	109	22 ± 0.74^a^	5.5 ± 0.44^c^	5 ± 0^b^	5 ± 0.50^b^	1.5 ± 0^c^				3 ± 0.45^c^	28.13
WamaT	0.14 ± 1.30	91	22.5 ± 0.90^a^	13 ± 0.86^b^	4 ± 0^c^	4 ± 0.58			2.5 ± 0.50^b^		4 ± 0.52^b^	64.52
Yoom	0.12 ± 1.06	101	19.5 ± 0.82^a^	12 ± 0.52^b^	6.5 ± 0.40^b^	4.5 ± 0^b^						9.68
Yoruba	0.05 ± 0.84	67	11 ± 0.72^b^		3 ± 0^c^			2.5 ± 0^b^	2 ± 0^b^			7.14
Zerma	0.08 ± 0.74	101	5.5 ± 0.40^c^	3.5 ± 0^c^	9 ± 0.56^a^	5 ± 0^b^		3 ± 0.45^b^				14.63
Means ± sd	0.10 ± 0.19	84	14.00 ± 0.73	7.50 ± 0.39	7.21 ± 0.32	3.61 ± 0.23	1.02 ± 0.13	1.38 ± 0.10	1.59 ± 0.40	0.29 ± 0	0.79 ± 0.40	30.88

*UV* Use value, *UVc* use value of category, *sd* standard deviation, *Ni* abundance/density expressed in number of tufts ha^−1^, *ICV* index of commercial value; a*b*c: means in a row non-connected by the same letters are significantly different (*p* < 0.05)

**Table 5:** Use values, density and commercial value of *C. tinctorium*

Ethnic groups	*UV* ± sd	*Ni*	*UVc* ± sd						ICV
			Medicinal	Food	Fodder	Dye	Cosmetic	Veterinary	
BaribaB	0.36 ± 1.39	127	56.5 ± 1.01^a^	17.5 ± 0.00^a^	3.5 ± 0.00^b^		3.5 ± 0.00^a^		51.28
BaribaK	0.27 ± 1.38	119	49.5 ± 1.15^a^	15 ± 0.00^b^				4 ± 0.89^a^	66.67
BaribaN	0.18 ± 1.21	53	32 ± 1.21^c^	14 ± 0.00^b^					35.71
Berba	0.14 ± 1.24	79	25.5 ± 1.20^c^	10.5 ± 0.00^c^					37.93
Biali	0.18 ± 1.17	78	26.5 ± 1.10^c^	19.5 ± 0.51^a^					45.16
Boko	0.22 ± 1.17	81	41 ± 1.36^b^	12.5 ± 0.00^c^			2 ± 0.00^b^		65.52
Dendi	0.21 ± 1.21	91	36.5 ± 1.38^b^	13.5 ± 0.00^b^		2.5 ± 0.00^b^			58.06
Gourmantché	0.26 ± 1.59	101	47.5 ± 1.44^a^	16.5 ± 0.35^a^				2.5 ± 0.50^b^	50.00
Haoussa	0.19 ± 1.12	81	30 ± 0.79^c^	14 ± 0.27^b^			4 ± 0.00^a^		44.83
Mokolé	0.33 ± 1.28	109	62 ± 1.11^a^	15 ± 0.00^b^		6 ± 0.55^a^			76.67
Natimba	0.26 ± 1.37	91	47 ± 1.33^a^	15 ± 0.44^b^	2.5 ± 0.00^c^				44.44
N'gnindé	0.20 ± 1.37	79	31.5 ± 1.05^c^	15.5 ± 0.40^b^		4.5 ± 0.55^b^			37.93
Peulh	0.14 ± 0.76	100	28.5 ± 0.81^c^	6.5 ± 0.00^c^	3 ± 0.00^b^				30.00
Gando	0.12 ± 1.31	61	25 ± 1.39^c^	6.5 ± 0.00^c^	4.5 ± 0.00^a^				47.06
WamaM	0.27 ± 1.37	96	49.5 ± 1.17^a^	14.5 ± 0.00^c^				3.5 ± 0.55^b^	37.50
WamaT	0.26 ± 1.59	87	44 ± 1.29^b^	17.5 ± 0.47^a^				4 ± 0.55^a^	75.97
Zerma	0.26 ± 1.06	213	47.5 ± 1.06^a^	17.5 ± 0.00^a^					87.80
Means ± sd	0.23 ± 0.20	97	40.00 ± 1.17	14.18 ± 0.14	3.38 ± 0.00	4.33 ± 0.37	3.17 ± 0.00	3.50 ± 0.62	52.21

*UV* Use value, *UVc* use value of category, *sd* standard deviation, *Ni* abundance/density expressed in number of tufts ha^−1^, *ICV* index of commercial value; a*b*c: means in a row non-connected by the same letters are significantly different (*p* < 0.05)

**Fig. 4:**
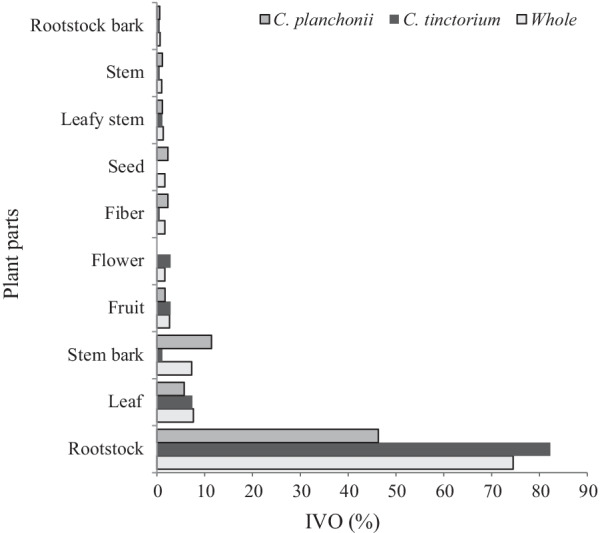
Index values of organs from *Cochlospermum* species

**Fig. 5:**
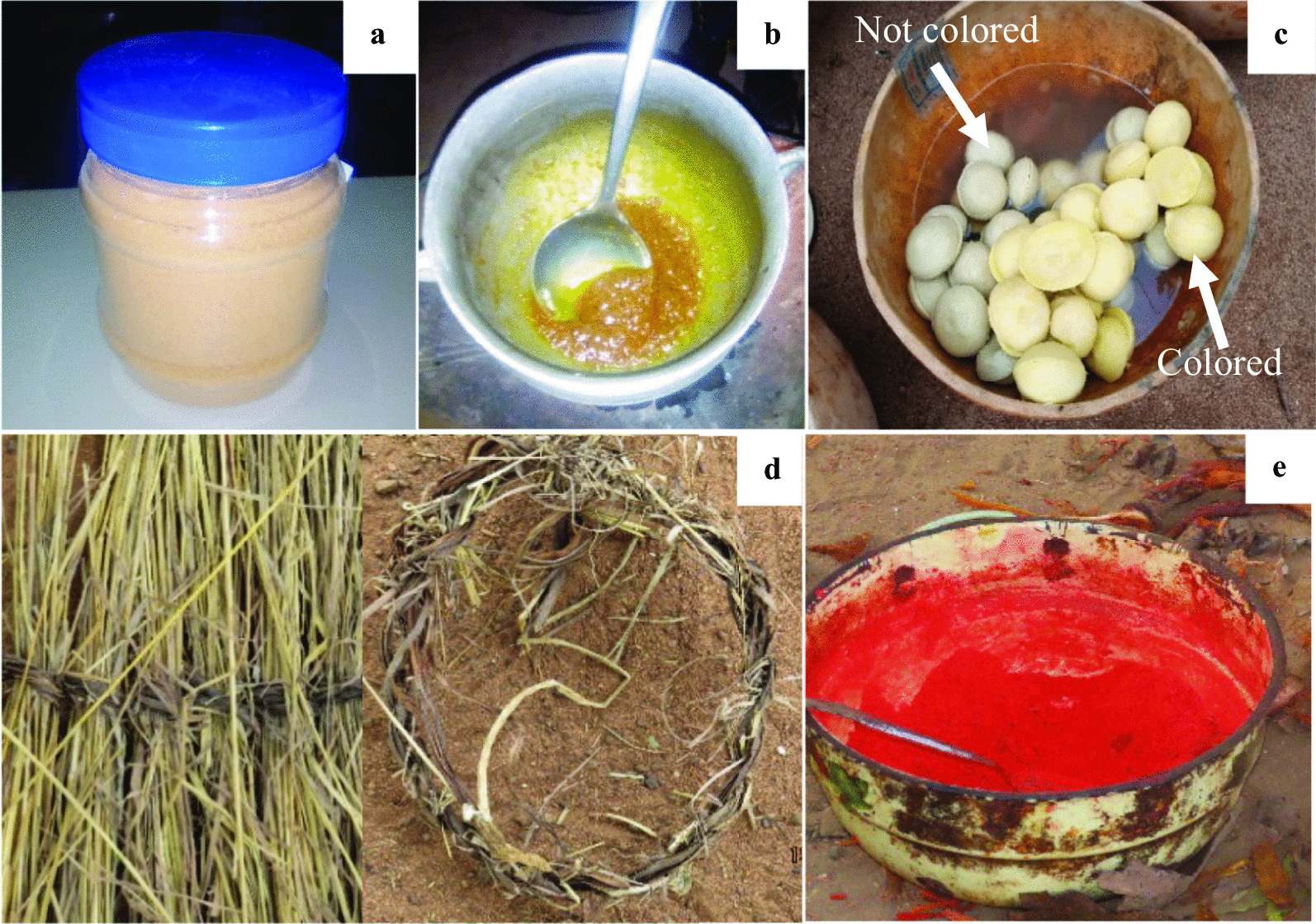
Some use-reports of* Cochlospermum* species in Benin.** a** Dry powder from* Cochlospermum* rootstock sold for medicinal purposes at Hospital of “Saint Jean de Dieu” (Tanguiéta municipality),** b** Sauce preparation with rootstock powder,** c** Yellowed shea butter with rootstock powder,** d** Rope made from stem bark of* C. planchonii*,** e** Additive used to further yellow* Cochlospermum* rootstock powder

Moreover, among the collected plant parts, rootstock was used by 74.50% of the populations regardless of the species, i.e., 82.29% for *C. tinctorium* and 46.29% for *C. planchonii* (Fig. 4). In contrast, *C. tinctorium* stems and fibers (0.57%) were weakly mentioned, while *C. planchonii* seeds and flowers were not reported by informants. This trend did not vary significantly across the three studied biogeographical zones studied either for *C. planchonii* or for *C. tinctorium*. As examples, the powder of rootstocks from both species was sold, either in plastic bottle (Fig. 5a) to treat hepatic affections, or traditionally used in sauce preparation (Fig. 5b), or to yellow shea butter (Fig. 5c). The stem of *C. planchonii* was used to thread rope (Fig. 5d) for various purposes.

#### Categories of uses and commercial importance

Overall, nine and six categories were reported, respectively, for *C. planchonii* (Table 4) and *C. tinctorium* (Table 5), of which medicinal use was the main category with fifty-four specific affections/symptoms treated, grouped into twelve categories (Table 6). Eighteen and eight of them were specifically reported for *C. tinctorium* and *C. planchonii*, respectively, and twenty-eight common to both. Jaundice (12.7% for *C. tinctorium* and 5.99% for *C. planchonii*) and malaria (10.32% for *C. tinctorium* and 4.79% for *C. planchonii*) were the most common affections. The main categories were general health category (28.3%), followed by gastrointestinal (15.09%) and infectious (16.98%) whatever the species. The medicinal use category was followed by food (7.50 ± 0.39) and handicraft (7.21 ± 0.32) categories for *C. planchonii* (Table 4), food uses (14.18 ± 0.14) and dye (4.33 ± 0.37) for *C. tinctorium* (Table 5). In contrast, fuel (0.29 ± 0) and cosmetic (3.17 ± 0) categories were the less reported, respectively, for *C. planchonii* and *C. tinctorium*.

**Table 6:** Categories and citation frequency of affections treated by *Cochlospermum* species

Categories (FC %)	Affections	Citation frequency (%)		
		Whole	*C. tinctorium*	*C. planchonii*
Dermatology (5.66%)	Abscess	0.60	–	0.60
	Dermatosis	1.20	0.79	0.60
	Edema	1.80	2.38	–
Eye (1.89%)	Eye diseases	1.20	1.59	–
Gynecology/andrology (5.66%)	Hernia	1.20	1.59	0.60
	Miscarriage	0.60	0.79	–
	Swollen testicle	0.60	0.79	0.60
General health (28.3%)	Body swelling	0.60	0.79	0.60
	Breast swelling	0.60	0.79	0.60
	Breast milk purification	0.60	0.79	0.60
	Child crisis	1.20	–	1.20
	Lower abdomen pain (after childbirth)	0.60	0.79	–
	Hemorrhoids	2.99	3.17	1.20
	Head ache	0.60	–	0.60
	Health promotion	0.60	0.79	–
	Immunodeficiency disorders	0.60	–	0.60
	Joint pain	1.80	2.38	–
	Knee pain	0.60	–	0.60
	Loss of appetite	0.60	0.79	0.60
	Lumbago	1.80	2.38	0.60
	Thinness	1.80	2.38	-
	Weakness	1.80	2.38	0.60
Gastrointestinal (15.09%)	Constipation	7.19	5.56	3.59
	Diarrhea	2.99	3.17	1.20
	Intestinal worm	1.20	1.59	0.60
	Purgative	7.19	4.76	3.59
	Rectorrhagia (stools bleeding)	1.20	0.79	0.60
	Stomach ache	2.99	3.17	1.80
	Stomach distending	0.60	0.79	–
	Ulcer	2.55	3.52	0.60
	Bloated stomach in children	0.35	0.35	–
Infectious (16.98%)	Cholera	0.60	0.79	–
	Hepatitis	0.60	0.79	0.60
	Malaria	10.78	10.32	4.79
	Measles	1.20	-	1.20
	Schistosomiasis	1.20	0.79	1.20
	Tetanus	0.60	0.79	0.60
	Tooth decay	3.59	3.97	0.60
	Whitlow	0.60	0.79	–
	Yellow fever	2.40	2.38	1.20
Liver (5.66%)	Diabetes	0.60	0.79	–
	Jaundice	11.98	12.70	5.99
	Lever diseases	1.80	2.38	1.20
Neurological (3.77%)	Nervous trouble	1.20	1.59	–
	Paralysis	0.60	0.79	–
Respiratory (1.89%)	Cough	0.60	0.79	–
Skeleto-muscular (3.77%)	Bone solidification	0.60	–	0.60
	Convulsion	0.60	0.79	0.60
Urological (1.89%)	Blennorrhoea	1.20	0.79	0.60
Unspecific (5.66%)	Burn	0.60	–	0.60
	Coldness	0.60	0.79	–
	Fever	2.99	2.38	1.80
Vascular/blood (3.77%)	Anemia	1.20	1.59	–
	High blood pressure	3.59	4.76	–

**Fig. 6:**
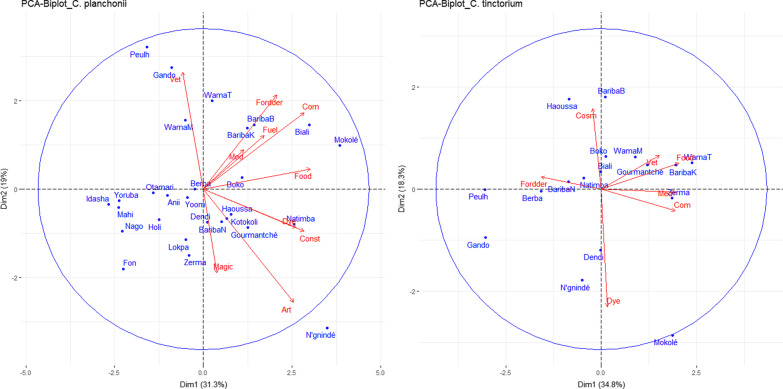
Projection of ethnic groups into the principal component analysis (PCA) using use categories of *C. planchonii* (left) and *C. tinctorium* (right).* Med* Medicinal,* Art* Handicraft,* Const* Construction,* Cosm* Cosmetic,* Vet* Veterinary,* Com* Commercial

The use values (UV) generally varied across the ethnic groups as well as within the same ethnic groups (Bariba and Wama), questioned in different geographical areas (Tables 4 and 5). Thus, the highest UVs for *C. planchonii* were recorded in Biali (0.18 ± 1.26), while the lowest were in Idasha (0.04 ± 0.89) and Yoruba (0.05 ± 0.84). Regarding *C. tinctorium*, Bariba’s of Banikoara (0.36 ± 1.39) and Mokolé (0.33 ± 1.28) showed the highest use values, while Peulh (0.14 ± 0.76) and Gando (0.12 ± 1.31) revealed the lowest. This use pattern was projected into the principal component space to grasp the link ethnic groups and the use categories (Fig. 6). It showed that WamaT, Mokolé, Biali, Boko, BaribaK and BaribaB used *C. planchonii* mainly for fodder, fuel, medicine, food and commercial purposes, while the same uses were mainly reported for Gourmantché, WamaT and BaribaK regarding *C. tinctorium*. If the ethnoveterinary uses of *C. planchonii* were mainly attributed to Gando, the uses of *C. tinctorium* for the same purpose were recorded in Gourmantché and Wama ethnic groups.

**Fig. 7:**
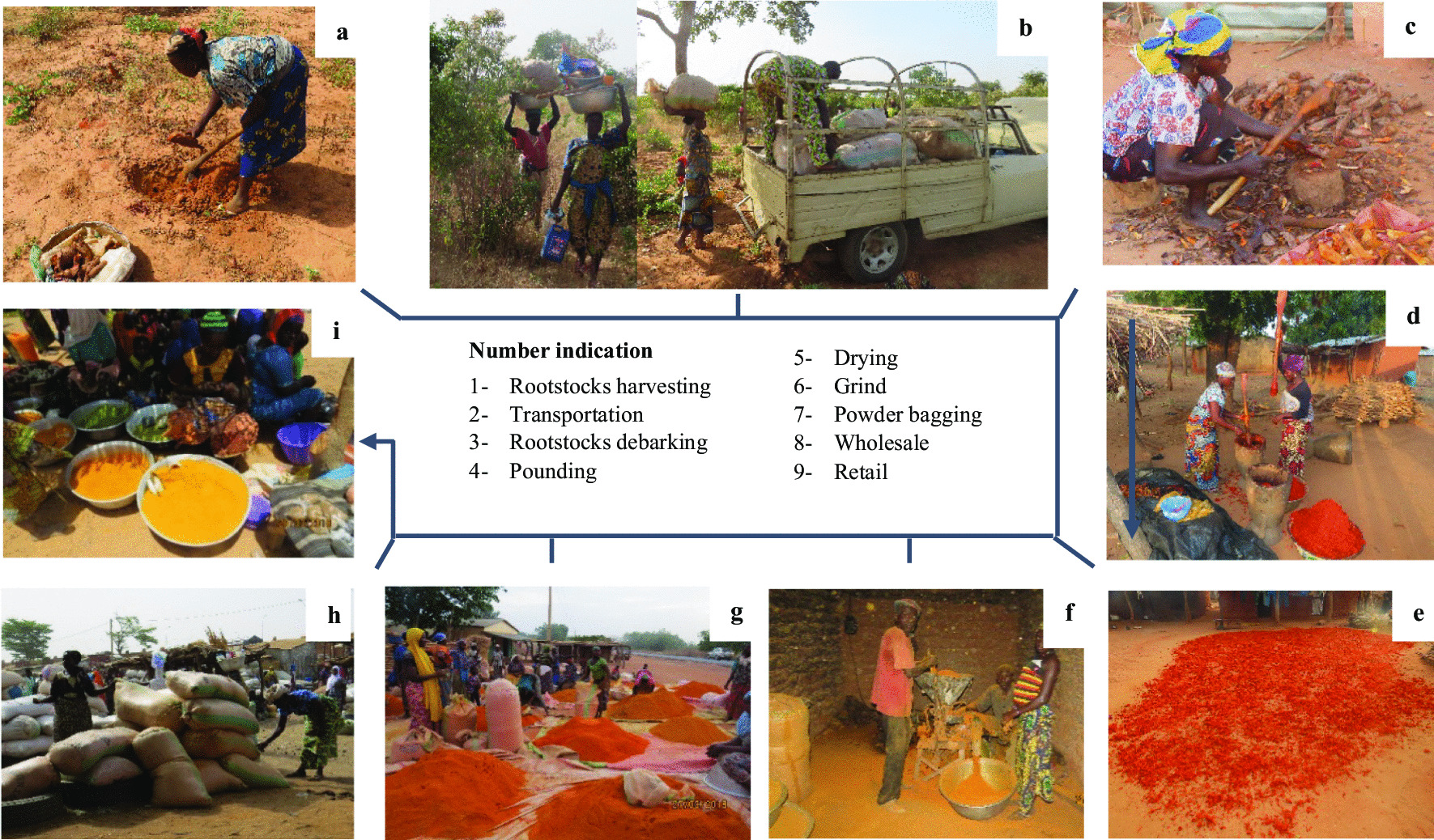
Process of powder production from *Cochlospermum *species rootstocks. **a** Rootstocks harvesting in the wild, **b**  Transportation at home, **c** Barking of rootstocks, **d** Crushing of rootstocks pieces, **e** Drying of crushed rootstock, **f** Crushing at the mill, **g** Bagging of roottcosk powder, **h** Wholesale market, **i** Retail market

Finally, the mean index of commercial values (ICV) reported for *C. planchonii* and *C. tinctorium* was 30.88% and 52.21%, respectively, with the highest ICV recorded in Mokolé (66.67%) and Zerma (87.80%), respectively, for *C. planchonii* and *C. tinctorium*. Thus, only *C. tinctorium* appeared as commercially important (ICV ≥ 75%) for Zerma, Mokolé and Wama ethnic groups (Table 5). This trade was mainly practiced by young and old women, which produced and sold rootstock powder in local markets, notably in Malanville, Natitingou and Tanguiéta municipalities, to either national or neighboring country (Nigeria, Niger and Burkina-Faso) customers, following a well-defined process (Fig. 7a–i). Broadly, about 42.3% of women producing the powder practiced it as principal activity, while more than 57% produced this powder at the end of farm work. However, due to market preferences, the yellow color of the raw powder was intensified using other products, such as shea butter or colorant (Fig. 5e).

#### Local abundance and its temporal change

The number of stem tufts recorded varied significantly across the areas, thus showing the abundance/availability of each species. For *C. tinctorium*, an average of 97 tufts.ha^−1^ was recorded in the study area with 53 tufts ha^−1^ as the lowest density in municipality of N’Dali in the Sudano-Guinean zone, and 213 tufts ha^−1^ as the highest, recorded in the municipality of Malanville in the Sudanian zone (Table 5). With a mean of 87 tufts ha^−1^ in the study area, the lowest density of *C. planchonii* was 31 tufts ha^−1^ recorded in the Fon ethnic group (municipality of Zakpota in the Sudano-Guinean zone), while the highest number was 139 tufts ha^−1^ in the Wama ethnic group (municipality Toucountouna in the Sudanian zone) (Table 4). Moreover, the temporal change in this local abundance is broadly considered to decline deeply countrywide. In fact, the majority (81.09% and 73.7% for *C. tinctorium* and *C. planchonii*, respectively) of informants reported a decrease in *Cochlospermum* population abundance. Contrary to that, 18.91% and 26.30% of informants perceived that abundance of *C. tinctorium* and *C. planchonii*, respectively, did not change, while no informant reported an increase in both species’ population abundance. This trend did not significantly vary (*p* > 0.05) whatever the phytogeographical zone as well as the species.

### Resources availability versus cross-cultural patterns hypotheses

Findings revealed that both species’ local abundance was significantly positively correlated with the use value recorded in municipalities studied. Thus, the Spearman’s correlation coefficients were, respectively, high for *C. tinctorium* (*r* = 0.76; *p* < 0.001) and moderate for *C. planchonii* (*r* = 0.45; *p* < 0.05). Therefore, the resource availability hypothesis explained the relationship between the local abundance of *C. tinctorium* and *C. planchonii* and their use values across the study area. Moreover, the abundance of *C. tinctorium* appeared positively significantly correlated with its commercial value (*r* = 0.64; *p* < 0.05), contrary to *C. planchonii* which local abundance did not show a correlation (*r* = 0.36; *p* > 0.05) with its commercial value.

On the other hand, the Kruskal–Wallis test performed revealed significant differences between the use values of *C. tinctorium* (*χ*^2^ = 135.43, d*f* = 21, *p* value < 0.05) and *C. planchonii* ((*χ*^2^ = 118.19, d*f* = 17, *p* < 0.05) across the ethnic groups studied. This means that the cultural patterns are also function of the use values of each of these species in the study area.

### Harvesting modes and conservation management practices

Given that the rootstock was the main plant parts exploited, the major mode of harvest consisted of uprooting the individuals’ species. For that, most of the informants reported to collect the rootstocks by digging with axe (51.05%), hoe (37.91%) and machete (11.04%). Thus, the axe was mainly used by rootstock powder producers (81.21%), followed by healers (13.94%), while the hoe was mainly used by healers (73.41%) and machete was used by occasional harvesters such as farmers and others (76.56%). In addition, informants experienced in rocky or hard soil work used mostly hatchet, while those experienced in thick or arable soil work usually used hoe.

**Fig. 8:**
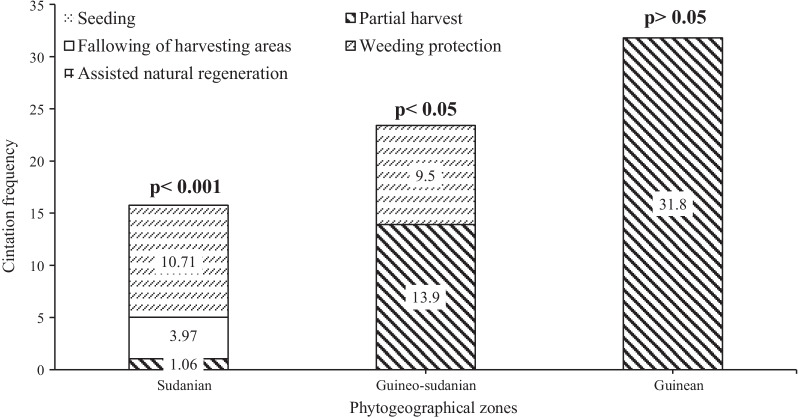
Local practices in *Cochlospermum* species conservation across the phytogeographical zones

On the other hand, three practices out of the five retained were practiced by informants (41.8% in Guineo-Congolian zone, 23.4% in Sudano-Guinean zone and 15.74% in Sudanian zone) for *Cochlospermum* species conservation. If seeding and assisted natural regeneration actions were absent, partial harvest of rootstocks (41.8%, 13.9% and 1.06%, respectively, in Guineo-Congolian, Sudano-Guinean and Sudanian zones), weeding protection (10.71 and 9.5% in the Sudanian and Sudano-Guinean zones) and fallowing of harvesting areas (3.97% only in Sudanian zone) were broadly practiced by few informant depending on the phytogeographical zone. Across the study area, only local populations of Angaradebou village (located in 11°19′44″N, 3°02′26″E; Kandi municipality in the Sudanian zone) practiced a near-common conservation strategies, consisting in partial harvest and fallowing of harvesting areas. These actions were initiated by the village wise men in collaboration with local foresters. The conservation practices varied significantly in the Sudanian (*p* > 0.001) and Sudano-Guinean (*p* < 0.05) zones, while it did not significantly vary in Guineo-Congolian zone (*p* > 0.05) (Fig. 8).

## Discussion

### Indigenous knowledge on *Cochlospermum* species

The findings in this study revealed that *C. tinctorium* and *C. planchonii* are well known and identified by various names, meaning their importance for rural communities. Although vernacular names recorded showed an excess of monotypic taxa, it also revealed a polytypic diversity, e.g., *Tchotcho'nda*, *Avokanfoun tchéké*, *Lissaya'nigou*, as globally reported on medicinal plant [53]. In addition, the meanings of the vernacular names were exclusively descriptive and conveyed the traditional uses such as food (e.g., *Boussorobu’nibou, Dissondi*), therapeutic (*Abuburoumey, Lissaya'nigou, Gbètou*) or cultural (*Tòòri, Tòòra*) purposes. This emphasized previous findings positing that the majority of folk nomenclatures of plants are mnemonics and reflect a broad spectrum of information on local uses, ecology, anatomy and several other aspects, which could differ greatly within peoples, areas and cultures [54–57]. Moreover, similarity was noted in vernacular names of different ethnic groups, e.g., Dendi and Zerma naming the both species as *Kpata*. This is likely due to the nearness of the two ethnic groups, on the point of view of geographical location, and also of their close relationship in history [58, 59]. In the past, similar observations have been reported within two ethnic groups in Mexico having common ancestors who share cognate plant names in their pharmacopeias [60].

Overall, 83 specific uses were recorded, grouped into ten categories, largely dominated by medicinal uses with 54 affections and symptoms treated, followed by food uses. The predominance of these two categories was previously reported for all the species of *Cochlospermum* genus [40] and specially for *C. tinctorium* and *C. planchonii* across the West Africa [24]. In addition, reporting the species as curing diseases such as malaria, hepatitis b and jaundice make them medicinally important plants, since malaria appears as the world's most important parasitic disease, with devastating consequences [61, 62]. Moreover, although ten categories were recorded in this study, out of the fourteen reported by Johnson-Fulton [40], it was the first time the cosmetic use category was mentioned for *Cochlospermum* species. Conversely, horticulture, hunting and gum categories were not documented in this study [40]. Our findings confirm the wide range of uses of *Cochlospermum* species across West African countries, such as Benin [23, 63, 64], Nigeria and Ivory Coast [65], Burkina Faso [66, 67], and elsewhere [35]. In addition, as found in this study, literature review has highlighted gastrointestinal and infectious as the most categories of affections treated by these species [24, 40], and this emphasized the good antibacterial evidence of *Cochlospermum* species [68, 69].

### Species availability and cultural use patterns

For their needs, local people sought different plant parts of *Cochlospermum* species, whose abundance varied across the study areas. In fact, species abundance revealed Sudanian and Sudano-Guinean trends, respectively, for *C. tinctorium* and *C. planchonii*, reflecting likely their ecological affinity to semiarid and arid regions [26, 37]. Broadly, not only the rootstocks were the main organs sought for most of the purposes but also showed an important commercial value, notably in the northern Benin [23, 63]. Indeed, the trade of *Cochlospermum* rootstocks powder employs many women of various ages who practice this business as a principal or secondary activity. Year-round, and mainly after the crop harvest, women harvest, powder abundant rootstocks, and sell in local markets, to enhance their household financial capacity. Although the economic importance of *Cochlospermum* species was scarcely documented, the trade around their rootstocks powder is highly helpful for rural populations, mainly in northern Benin [64]. Therefore, *Cochlospermum* species could be considered as good candidates for commercial production regarding their economic evidence [70, 71].

As mentioned by informants, the *Cochlospermum* species were increasingly used not only because of their useful rootstocks, but also because of their availability year-round, even in dry seasons when most of the plant resources are scarce and previous cropping is depleted [72]. Therefore, food uses are more accentuated to compensate for the lack or the expense of tomatoes and palm oil [64, 73], thus increasing demand in the market. On the one hand, findings in this study revealed that people in northern Benin, where species were more abundant, used more both the species than these in center and south where species were less abundant. By that, the species abundance was correlated with their use values, supporting the resource availability hypothesis. This hypothesis has been previously tested in different geographical areas in Africa [74] and elsewhere [5, 12, 14, 75] to assess the close relationships between relative importance (measured by its use value) and a plant's local availability/abundance. On the other hand, it was also proved that ethnic groups were closely correlated with the use value of the both species, supporting the cultural importance in *Cochlospermum* species’ uses in Benin as reported for other plant species [45, 76–78].

However, although both hypotheses of this study were confirmed, it is important to consider other factors that can considerably influence the analyses performed and the findings. If in the first time, if the use values of populations in the north were the most important, it can be due to the very low purchasing power of most households in this region. Indeed, the populations of northern Benin are essentially rural and agricultural and therefore have a relatively low financial capacity [33]. Thus, the high consumption of wild plants is not necessarily due to their local abundance, but rather to an alternative linked to the economic situation and therefore to low purchasing power. Moreover, the geographical location of the ethnic groups surveyed may also be an important factor. In this study, the ethnic groups with the highest use values were essentially located in the north part of Benin. Therefore, it appeared important to consider other factors in analysis performed for these hypotheses in order to enhance their veracity.

### Implications for conservation

As reported in study, there is a growing concern about declining populations of *Cochlospermum*, as reported by the majority of informants. In fact, they considered conservation actions unnecessary, since both species regenerate naturally in the wild. Unfortunately, the harvesting mode adopted by several informants was to dig up and remove the rootstocks entirely. This practice causes killing the plant individual, and by that a considerable diminishing plant populations that likely lead to local extinction of the target species [79]. According to Leso et al. [80], these practices do not seem to ensure sustainable management and thus long-term availability of the species. As evidence, some harvesters confirmed having to go much further to harvest *Cochlospermum* plant parts, like the women of Banitè village (Malanville commune, located in Sudanian zone, at 11°52′00″N, 3°23′00″E), who are forced to rent cars (Fig. 7b) to collect large quantities of rootstocks from neighboring localities.

Yet, some authors have already pointed out the overuse of *Cochlospermum* species for various purposes and underlined the pressure they are facing [79, 81, 82]. As posited, the high use value of plant species experiences them to a harvesting pressure [83], and hence the need for their sustainable management policies [20]. Therefore, if current economic, nutrient and cultural contribution of *Cochlospermum* species in rural populations’ well-being could be maintained and enhanced, sustainability approaches must be taken into account. Indeed, meeting sustainable management implies to overcome the current needs without compromising the ability of future generations to meet their own needs [84]. A great challenge must be taken up in the context of a growing market demand for *Cochlospermum* root powder, leading to an increase in the quantities of roots harvested by the producers. In addition, there is a strong anthropic pressure linked to several causes of vegetation degradation of which deforestation, fire as a tool for hunting, and clearing for installing new or extending existing agricultural lands [85]. Much progress has made it possible to lead people to free oneself with wild food owing to agricultural expansion; nevertheless, this strongly contributes to ecosystem degradation and biodiversity loss [22].

Although rural populations are sometimes pioneers in the domestication process [64], no seeding or assisted natural regeneration was practiced by informants in this study. However, producers affirmed they will welcome any initiative of large-scale reproduction of the target species, so that to have much more amount *Cochlospermum* rootstocks. Therefore, future investigations are needed on the impact of harvesting on the regeneration capacity of rootstocks, the biomass of rootstocks produced over a period of time, and the impact of harvesting on the population dynamics of these species. In addition, given the important economic, cultural and diet roles of *Cochlospermum* species, and the threats they are faced, they need to be considered within the priority species for conservation issues [45, 70]. For this time, existing local conservation practices, such as fallowing of harvesting areas and partial harvest, should be popularized, while others, such as seedlings and assisted natural regeneration, must be initiated by the local harvesters.

Furthermore, in the context of species conservation, the assessment of species diversity remains one of the major and key steps in conservation biology [86, 87]. Although diversity within the genus *Cochlospermum* in Benin is not yet elucidated, this study prioritizes the assessment of use patterns and local management of two well-known species, namely *C. planchonii* and *C. tinctorium*, to provide tangible data for their sustainable management [86]. In addition, morphological variations were noted in the collected vouchers of *Cochlospermum* species in this study. Therefore, it will be very interesting to determine the boundaries of *Cochlospermum* species based on morphological and reproductive variations [24]. The findings of this study will undoubtedly be of great value in filling the current gaps in species diversity within the West African genus *Cochlospermum* and thus contribute to improving biodiversity countrywide and in the sub-Saharan region [88].

## Conclusion

*Cochlospermum* species are well-known species widely used for multipurpose in Benin. The use purposes reported varied significantly countrywide and were grouped into ten categories, including food, medicine, veterinary, dye, fuel, construction, cosmetic, fodder, magic and handicraft uses. The medicine uses were the main with 54 affections treated. The use values recorded were significantly correlated with the local abundance as well as the ethnic groups, proving the resource availability and cross-cultural patterns hypotheses. The rootstocks were the main plant parts sought and were highly sold as powder, giving rural women a financial income. However, the decline in both species populations becomes a growth concern in *Cochlospermum* species sustainability. In fact, conservation actions were practiced by very few people which consisted of the partial harvest, fallowing of the harvesting areas. In the face of this depletion, actions are needed to introduce the large-scale propagation of *Cochlospermum* species and by that to ensure the sustainable availability of these important wild edible plants. Finally, promotion of existing conservation strategies and introduction of additional strategies such as seeding, weed protection and assisted natural regeneration will contribute to the sustainable use of *Cochlospermum* species.

## Data Availability

The datasets used and/or analyzed in this study are available from the corresponding author on reasonable request.

## Abbreviations

DPs: Dialog Partners
EDSVT: École Doctorale Sciences de la Vie et de la Terre
FC: Frequency of citation
INSAE: Institut National de la Statistique et de l’Analyse Économique
ISE: International Society of Ethnobiology
PCA: Principal component analysis
RAH: Resources availability hypothesis
UAC: University of Abomey-Calavi
VU: Use value
WEPs: Wild edible plants
